# Ghee Butter from Bovine Colostrum Reduces Inflammation in the Mouse Model of Acute Pancreatitis with Potential Involvement of Free Fatty Acid Receptors

**DOI:** 10.3390/nu13093271

**Published:** 2021-09-19

**Authors:** Aleksandra Tarasiuk, Marcin Talar, Kamila Bulak, Jakub Fichna

**Affiliations:** 1 Department of Biochemistry, Faculty of Medicine, Medical University of Lodz, 92-215 Lodz, Poland; tarasiuk.aleksandra@gmail.com (A.T.); marcin.talar@umed.lodz.pl (M.T.); 2 Department of Pathomorphology and Forensic Veterinary Medicine, Faculty of Veterinary Medicine, University of Life Sciences in Lublin, 20-612 Lublin, Poland; kamila.bulak@up.lublin.pl

**Keywords:** acute pancreatitis, L-arginine-induced acute pancreatitis, ghee butter from colostrum, colostrum, inflammation

## Abstract

Acute pancreatitis (AP) is an inflammatory disease that causes severe tissue damage. Ghee butter from bovine colostrum (GBBC) is a clarified butter produced by heating milk fat to 40 °C and separating the precipitating protein. As colostrum mainly contains fatty acids (FAs), immunoglobulins, maternal immune cells, and cytokines, we hypothesized that it may exert anti-inflammatory effects. We investigated the effects of GBBC on experimental AP in mice. Two intraperitoneal (*ip*) injections of L-arginine (8%) were given 1 h apart to generate the AP murine model. After 12 h from the first L-arginine injection, mice were divided into the following experimental groups: AP mice treated with GBBC (oral gavage (*po*) every 12 h) and non-treated AP mice (*po* vehicle every 12 h). Control animals received vehicle only. At 72 h, mice were euthanized. Histopathological examination along with myeloperoxidase (MPO) and amylase/lipase activity assays were performed. In a separate set of experiments, FFAR1 and FFAR4 antagonists were used to verify the involvement of respective receptors. Administration of GBBC decreased MPO activity in the pancreas and lungs along with the microscopical severity of AP in mice. Moreover, treatment with GBBC normalized pancreatic enzyme activity. FFAR1 and FFAR4 antagonists tended to reverse the anti-inflammatory effect of GBBC in mouse AP. Our results suggest that GBBC displays anti-inflammatory effects in the mouse model of AP, with the putative involvement of FFARs. This is the first study to show the anti-inflammatory potential of a nutritional supplement derived from GBBC.

## 1. Introduction

Inflammation is one of the most common pathological disorders affecting the pancreas [1,2,3]. There are three types of pancreatitis—acute pancreatitis (AP), chronic pancreatitis (CP), and hereditary pancreatitis. In AP, the local and systemic inflammatory reaction is the result of the interaction between activated endothelial cells and leukocytes, platelets, and mast cells, and released mediators. An inadequate and inappropriately directed inflammatory response leads—in the form of systemic inflammatory response syndrome (SIRS) and coagulopathy—to the development of organ failure. Prevention of AP is based primarily on starting treatment when any abnormalities related to pancreas functioning are detected. Importantly, AP is one of the factors that increase the risk of developing CP [1]. Moreover, even after 10 years of follow-up, patients hospitalized with AP have a twofold greater risk of developing pancreatic cancer compared to the general population [4].

Ghee butter (clarified butter) from bovine colostrum (GBBC) is made from a natural product of the mammary glands (formed in the last stage of pregnancy up to several hours after childbirth, when it is gradually transformed into milk) and has been recommended for culinary use and as a nutritional supplement. The biological role of GBBC has never been verified in the pathophysiology of AP, but the properties of both clarified butter and colostrum may suggest its anti-inflammatory effect [5,6,7,8]. Namely, colostrum has immunomodulatory (mainly attributed to lactoferrin and proline-rich polypeptide, PRP) [9,10,11], antibacterial [11,12,13], antiviral (based on the interaction of lactoferrin with both viral particles and their receptors on the surfaces of target cells) [9,12,13,14], antifungal (associated with damage to the cellular or mitochondrial membrane of the *Cryptococcus neoformans* and *Candida albicans* strains) [9,15], and antiparasitic [16] properties. According to some authors, casein contained in colostrum has the ability to protect against the onset of diabetes while weakening the likelihood of autoimmune reactions and stimulating the formation of new pancreatic islets [17,18,19].

The principal sources of free fatty acids (FFAs) include long- and medium-chain fatty acids drawn mostly from dietary triglycerides, as well as short-chain fatty acids (SCFAs) produced by the gut microbial fermentation of otherwise indigestible food fiber. FAs act as natural ligands for a group of G-protein-coupled receptors (GPCRs) that are called free fatty acid receptors (FFARs). FFAR1 and FFAR4 are activated by long-chain saturated and unsaturated FAs, while FFAR3 and FFAR2 are activated by SCFAs, mainly acetate, butyrate, and propionate. FFARs have been shown to mediate a variety of physiological and pathophysiological functions, including insulin and incretin hormone secretion facilitation, adipocyte differentiation, and modulation of inflammation, neuronal responses, and taste preferences [20]. Analyses showed that GBBC is an important source of FFAs, including saturated FAs such as oleic acid.

The anti-inflammatory effect of GBBC was tested in this study using the mouse model of L-arginine-induced AP, which is well known to mimic clinical AP and is commonly used to assess the impact of new potential treatment options [21,22,23]. Furthermore, we attempted to characterize the probable mechanism of action of GBBC in AP via FFARs.

## 2. Materials and Methods

### 2.1. Animals

Male C57BL/6 mice, 8–12 weeks old, were purchased from the University of Lodz’s Animal House in Lodz, Poland. All the animals were kept in normal rodent shoebox cages in a temperature-controlled environment with a 22 °C ambient temperature and a 12:12-h light–dark cycle. Animals were fed normal laboratory food and allowed free access to water. They were then divided into control and experimental groups (*n* = 8–12). All animal protocols were approved by the Lodz Animal Care Committee (#39/LB178/2020). All attempts were made to minimize animal suffering and to reduce the number of animals used.

### 2.2. Induction of Acute Pancreatitis

A sterile solution of L-arginine monohydrochloride (8%) was prepared in 0.9% NaCl, and the pH was adjusted to 7.0. At the beginning of the experiment, nonfasted mice were given a 4 g/kg body weight (BW) dose intraperitoneally (*ip*), after which the animals were returned to their cages and given free access to food and water. After 1 h, the animals received a second dose of L-arginine monohydrochloride solution (4 g/kg BW) in saline. The controls were given a sham injection of 0.9% NaCl only. Mice were weighed daily, and clinical symptoms of AP were observed. Mice were euthanized 72 h after receiving the second injection of L-arginine monohydrochloride solution or vehicle [21].

### 2.3. Treatment with GBBC

As GBBC has a solid state, it was heated to 40 °C prior to intragastric administration through a stainless-steel bulb-tipped gavage needle. Three doses of GBBC were selected: 1.10 µL of GBBC + 90 µL of olive oil, used as solvent (10% GBBC *v*/*v*), 2.50 µL of GBBC + 50 µL of olive oil (50% GBBC *v*/*v*), and 3.100 µL GBBC (100% GBBC *v*/*v*).

### 2.4. FFAR1 and FFAR4 Antagonist Administration

FFAR1 (DC 260126, Tocris, Bio-Techne, Warsaw, Poland) and FFAR4 (AH 7614, Tocris, Bio-Techne, Warsaw, Poland) antagonist solutions in 5% dimethyl sulfoxide (DMSO) were administered *ip* to nonfasted mice at a dose of 5 mg/kg BW 15 min before administration of GBBC (*po* 100%). Control mice received *ip* 5% DMSO vehicle solution.

### 2.5. Blood Serum and Tissue Collection

Mice were deeply anesthetized with a solution of ketamine (100 mg/kg BW) and xylazine (23.32 mg/kg BW) in 0.9% NaCl. Blood samples were collected from the venous sinus into 2 mL Eppendorf tubes and allowed to clot for 30–60 min at 37 °C. Further, the clot was left for at least 1 h or overnight at 4 °C to allow it to contract. Next, the serum was removed from the clot and centrifuged (10,000× *g* for 10 min at 4 °C) to remove any remaining insoluble material. Vials with serum were immediately frozen at −80 °C until further use. Following euthanasia, the entire pancreas and lungs from each animal were removed and immediately frozen in liquid nitrogen and kept at a temperature of 80 °C until needed.

### 2.6. Determination of Myeloperoxidase Activity

The MPO activity assay, as previously described, was used to monitor the degree of inflammation [24]. Tissue sections were weighed and homogenized in HTAB buffer (final concentration—50 mg tissue/1 mL HTAB buffer) using a Precellys Evolution advanced tissue homogenizer (Bertin Instruments, Montigny-le-Bretonneux, France) shortly after isolation. Homogenates were centrifuged (13,200× *g*, 15 min, 4 °C), and 7 μL of supernatant was added to each well on a 96-well plate, containing 200 μL of 500 mM potassium phosphate buffer (pH 6.0), supplemented with 0.167 mg/mL of O-dianisidine hydrochloride and 0.05 μL of 1% hydrogen peroxide. After 30 and 60 s, the absorbance was measured at 450 nm optical density (OD) (iMARK Microplate Reader, Biorad, Watford, UK). MPO was measured in milliunits per gram of wet tissue, with 1 unit corresponding to the amount of enzyme capable of converting 1 mol of H_2_O_2_ to water in 1 min at room temperature (RT). MPO activity was measured in units (U) per minute using a standard curve and purified peroxidase enzyme. Every measurement was taken in triplicate.

### 2.7. Determination of Amylase Activity in Blood Serum

The Amylase Assay Kit (Colorimetric) (Abcam, ab102523) was used to quantify α-amylase activity through a two-step reaction. In the protocol, the substrate ethylidene-pNP-G7 was cleaved by α-amylase to produce smaller fragments, which were then modified by α-glucosidase. This resulted in the release of a chromophore with an OD 405 nm. Accordingly, blood serum samples prepared according to manufacturer’s protocol, as well as standards and reaction mix, were added to the wells. Then, the samples were analyzed with a microplate reader every 2–3 min for 30–60 min at OD 405 nm. All measurements were performed in triplicate. Amylase activity in the test samples was calculated as:

$$\mathrm{Amylase}\;\mathrm{activity}=\left(\frac{B}{\Delta T\times V}\right)*D$$

where:

*B* = nitrophenol amount from the standard curve (in nmol);
Δ*T* = reaction time (T2–T1) (min);
*V* = pretreated sample volume added to the reaction well (in mL);
*D* = sample dilution factor.

Amylase = nmol/min/mL = mU/mL; 1 Unit Amylase = amount of amylase that cleaves ethylidene-pNP-G7 to generate 1.0 µmol of nitrophenol per min at pH 7.2 at 25 °C.

### 2.8. Determination of Lipase Activity in Blood Serum

Lipase activity was determined using the Lipase Activity Assay Kit (Colorimetric) (Abcam, ab102524), which hydrolyzed a triglyceride substrate to generate glycerol, which was measured by monitoring a change in the OxiRed™ probe (peroxidase fluorogenic substrate) absorbance (OD 570 nm). Accordingly, blood serum samples prepared according to the manufacturer’s protocol, as well as standards and reaction mix, were added to the wells. Then, the samples were analyzed with a microplate reader every 2–3 min for 60–90 min at 37 °C, protected from light at OD 570 nm. All measurements were performed in triplicate. Lipase activity in the tested samples was calculated as:

$$\mathrm{Lipase}\;\mathrm{activity}=\left(\frac{B\times D}{\Delta T\times V}\right)$$

where:

*B* = amount of glycerol in sample well calculated from standard curve (nmol);
Δ*T* = reaction time (T2–T1) (min);
*V* = original sample volume added into the reaction well (mL);
*D* = sample dilution factor.

Lipase = nmol/min/mL = mU/mL; 1 Unit Lipase = amount of lipase that hydrolyzes triglyceride to generate 1.0 µmol of glycerol per min at pH 7.2 at 37 °C.

### 2.9. Serum Blood Tests

Further serum blood sampling was performed using the Mindray BS-120 chemistry analyzer (Mindray Medical, Warsaw, Poland), focusing on the control group, AP mice, and AP mice treated with 100% GBBC. The following parameters were examined: glucose, creatinine, urea, total protein, alanine transaminase, aspartate transaminase, creatinine kinase, and calcium. In addition, to ensure the reliability and accuracy of test results, amylase and lipase were also quantified.

### 2.10. Western Blot Analysis

Samples of mouse pancreas tissue were mixed with the lysis buffer (50 mM Tris−HCl, pH 7.5; 300 mM NaCl; 1% Triton™ X-100; Sigma-Aldrich, Poznan, Poland), which contained protease inhibitor cocktail (4-(2-aminoethyl) benzene-1-sulfonyl fluoride at 104 mM, aprotinin at 80 μM, bestatin at 4 mM, E-64 at 1.4 mM, leupeptin at 2 mM and pepstatin at 1.5 mM). Samples were then homogenized using the Precellys^®^ Evolution Homogenizer (Bertin Instruments, Montigny-le-Bretonneux, France). After centrifugation of the homogenate (15,000× *g* for 15 min at 4 °C), the total protein concentration in each sample (triplicate) was determined using the Pierce™ 660 nm Protein Assay Reagent (Thermo Scientific, Rockford, IL, USA). To perform the electrophoresis of the samples, 4–20% Mini-PROTEAN^®^ TGX™ Precast Protein Gels (Bio-Rad, Warsaw, Poland) and electrophoretic buffer (0.1% SDS, 192 mM glycine, 25 mM Tris-base, pH 8.3) were used. After separation, proteins were transferred onto PVDF membranes (pore size: 0.45 μm; Life Technologies, Carlsbad, CA, USA) with the use of a semi-dry system with transfer buffer containing 20% (*v*/*v*) methanol, 192 mM glycine, 25 mM Tris-base, pH 8.3. The following conditions were applied for the incubation of the PVDF membranes: RT for 1 h in 5% non-fat dry milk in phosphate-buffered saline (PBS) with Tween^®^ 20 detergent (PBST; PBS, 0.1% Tween^®^ 20 detergent) to saturate non-specific protein binding sites. Next, for immunodetection of the proteins of interest, incubation of membranes with specific primary antibodies diluted in 1% non-fat dry milk in PBST was conducted for 80 min at RT. The following antibodies were used: primary rabbit anti-mouse FFAR1 polyclonal antibody (AFR-001; dil. 1:1000; Alomone Labs, Jerusalem, Israel), FFAR2 polyclonal antibody (AFR-032, dil. 1:1000; Alomone Labs, Jerusalem, Israel), FFAR3 polyclonal antibody (PA5-97747; dil. 1:1000; Thermo Scientific, Rockford, IL, USA), FFAR4 polyclonal antibody (E-AB-31,576; dil. 1:1000; Elabscience, Wuhan, China), and mouse beta actin (β-actin; sc-47778; dil. 1:1000; Santa Cruz Biotechnology, Dallas, TX, USA). Next, membranes were washed with PBST (5 times, 3 min) and incubated with appropriate horseradish peroxidase (HRP)-conjugated secondary antibodies for 1 h at RT. Then, the visualization of bands was performed using Super Signal West Pico Western Blotting Substrate (Thermo Scientific, Rockford, IL, USA) as a substrate for the localization of HRP activity. Analysis (qualitative and quantitative) was performed by measuring integrated OD using the ImageLab 6.1, ©2020, Bio-Rad Laboratories, Inc. for MacOS program (Bio-Rad, Warsaw, Poland). Molecular protein weight was determined using 5 μL/lane of Precision Plus Protein Standards (Bio-Rad, Warsaw, Poland).

### 2.11. Histopathological Analysis

Tissue samples from pancreas were fixed in 4% buffered formalin, pH 7.2, for 24 h before being processed automatically in a tissue processor (Leica TP-1020). Paraffin-embedded tissue samples were sectioned, mounted on slides, and stained with hematoxylin and eosin (H&E) according to normal protocol [25]. Masson’s trichrome staining was performed as described earlier [26]. Additionally, immunohistochemical reaction was conducted to detect α-smooth muscle actin in individual cells of exocrine pancreas. The primary anti-mouse α-SMA polyclonal antibody (ab5694; dil. 1:200, Abcam) was used. The immunohistochemical reaction was performed based on the indirect immunoperoxidase method using the ImmPRESS^®^ Universal Polymer Kit Peroxidase detection system (Horse Anti-Mouse/Rabbit IgG, Vector Laboratories, Burlingame, CA, USA). TBS (Tris-buffered saline) was used instead of the primary antibody to obtain the negative control for the immunohistochemistry (IHC) test. The positive tissue control for the primary antibody was mouse mammary gland. Nikon’s Eclipse E600 light microscope (Nikon Instruments Inc., Tokyo, Japan) was used to examine the pancreas samples. Images were taken with a digital imaging system that included a microscopy digital camera (Nikon DS-Fi1, Nikon Instruments Inc., Tokyo, Japan) and image analysis software (NIS-Elements BR-2.20, Laboratory Imaging, Praha, Czech Republic). A pathologist who was not aware of the experimental protocol performed the morphological analyses.

### 2.12. Drugs

Unless otherwise noted, all reagents were purchased from Sigma-Aldrich (Poznan, Poland). GBBC was a kind gift from P.W. “PROSZKI MLECZNE” (Nakło nad Notecią, Poland).

### 2.13. Statistical Analysis

Statistical analyses were performed using GraphPad PRISM 9.1.0 (GraphPad Software Inc., La Jolla, CA, USA). In the experiments, n denoted the number of individual outcomes that represented different animals. Normal distribution of data was verified with the Shapiro–Wilk test. Variance homogeneity was tested with Levene’s test. Results were expressed as box plots: mean ± SEM with min and max range for normally distributed variables or as median ± interquartile range (IQR: lower (25%) to upper (75%) quartile) with min and max range otherwise. For multiple comparisons, a one-way analysis of variance with Dunnett’s correction was employed to determine the significance of differences between groups with distributions not departing from normality. In the case of unequal variances, Welch’s correction was applied accordingly. When the normality assumption was violated, the significance of differences was tested with the Kruskal–Wallis test followed by post hoc Dunn’s test. The heat map showing the relationship between various metabolic enzyme activities measured in healthy mice, acute pancreatitis (AP) mice, and AP mice after administration of GBBC was shown as mean values. *p* values < 0.05 were considered statistically significant.

## 3. Results

### 3.1. Administration of GBBC Attenuated Pancreatitis in L-Arginine-Induced AP in Mice

**Figure 1:**
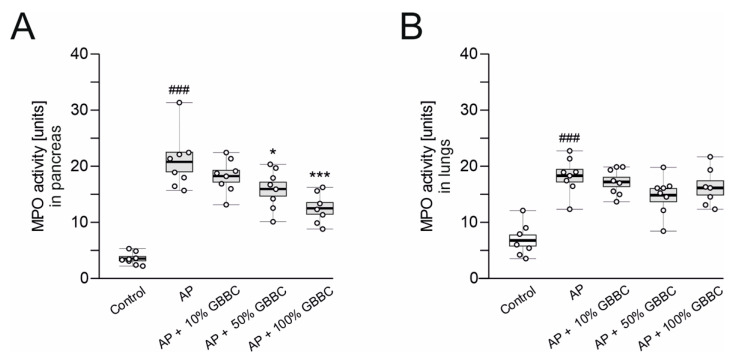
MPO activity in pancreas (**A**) and lungs (**B**) of healthy mice, mice with evoked acute pancreatitis (AP), and mice with AP after administration of increasing concentration of ghee butter from bovine colostrum (GBBC). Data presented as raw data (circles) and means (horizontal bolded lines) with SEM (boxes). The significance was calculated using one-way ANOVA and Dunnett’s post hoc test. *n* = 7–8; # (hash) denotes comparison to control. * (asterisk) indicates comparison to AP (acute pancreatitis). * denotes *p* < 0.05; ###/*** denotes *p* < 0.001.

Neutrophil sequestration in the pancreas is associated with the development of AP. As a result, because MPO activity in pancreatic tissues is a standard measurement of neutrophil infiltration, it has been used as a biochemical marker of AP. MPO activity in the lungs was used as a marker of systemic inflammation caused by proinflammatory cytokines during AP. Mice treated only with L-arginine had highly significantly increased MPO activity in the pancreas and lungs after 72 h compared with the control group (pancreas: 20.80 ± 1.76 vs. 3.54 ± 0.38 U, *p* < 0.001, Figure 1A; lungs: 18.30 ± 1.12 vs. 6.77 ± 0.99 U, *p* < 0.001, Figure 1B). Administration of 100% GBBC (po, 100 µL/mouse) significantly reduced MPO activity in the pancreas compared with AP mice (12.5 ± 1.05 vs. 20.80 ± 1.76 U, respectively, *p* < 0.001, Figure 1A). Administration of 50% GBBC also decreased MPO activity in the pancreas versus control group, but less potently than the higher concentration (15.8 ± 1.24 vs. 20.80 ± 1.76 U, respectively, *p* = 0.025, Figure 1A). Both concentrations of GBBC decreased MPO activity in the lungs, but the effect was non-significant. GBBC at the concentration of 10% had no significant effect on MPO activity, either in the pancreas or the lungs (Figure 1A,B, respectively).

### 3.2. Administration of GBBC Significantly Reduced Lipase Activity but Not Amylase Activity in the Blood Serum in L-Arginine-Induced AP in Mice

**Figure 2:**
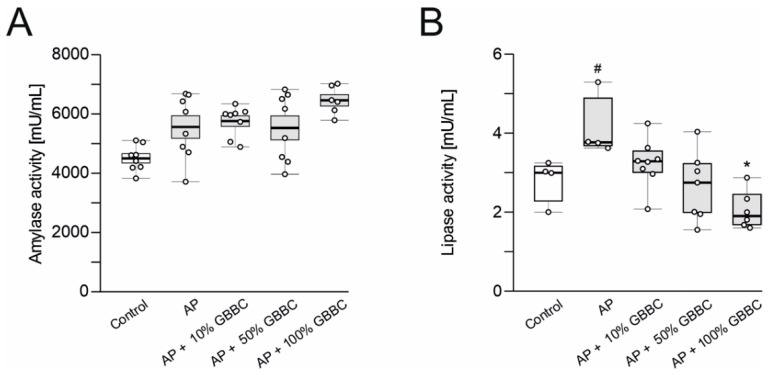
Amylase activity (**A**) and lipase activity (**B**) in the blood serum of healthy mice, mice with evoked acute pancreatitis (AP), and mice with AP after administration of increasing concentrations of ghee butter from bovine colostrum (GBBC). Data presented as raw data (circles) and means (horizontal bolded lines) with SEM (boxes) (**A**) or median with IQR (B). Significance estimated with the use of one-way ANOVA followed Dunnett’s post hoc test or with the use of the Kruskal–Wallis test with Dunn’s post hoc test for data departing from normal distribution. *n* = 4–8; # (hash) denotes comparison to control. * (asterisk) indicates comparison to AP (acute pancreatitis). #/* denotes *p* < 0.05.

Elevated plasma amylase and lipase are important markers of pancreatic acinar cell injury [27,28]. Administration of L-arginine increased amylase and lipase activity in the blood serum compared to controls (amylase: 5563 ± 380 vs. 4506 ± 154 mU/mL, Figure 2A; lipase: 4.11 ± 0.39 vs. 2.82 ± 0.28 mU/mL, *p* = 0.025, Figure 2B). Administration of GBBC, irrespective of concentration, did not reduce the amylase activity in the blood serum. Administration of GBBC concentration-dependently reduced lipase activity in the blood serum compared to AP mice, with a statistically significant effect observed at the concentration of 100% (2.05 ± 0.20 vs. 4.11 ± 0.40 mU/mL for 100% GBBC vs. AP mice, respectively, *p* = 0.021, Figure 2B).

### 3.3. GBBC Normalized Various Metabolic Enzyme Activities Increased in L-Arginine-Induced AP in Mice

**Figure 3:**
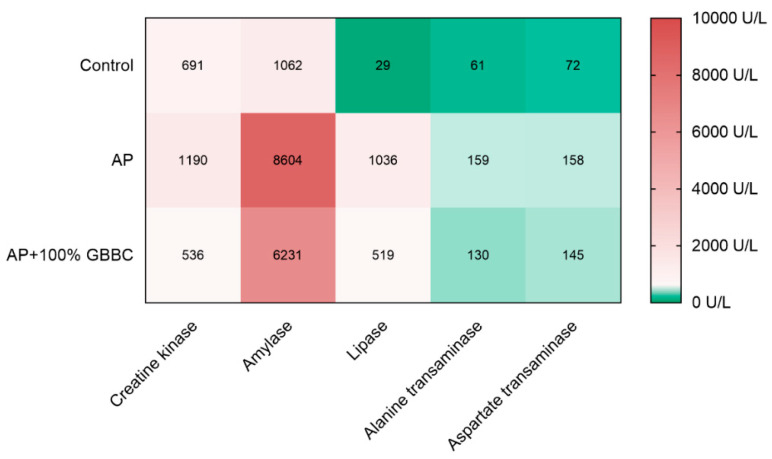
Heat map showing the relationship between various metabolic enzyme activities measured in healthy mice, mice with evoked acute pancreatitis (AP), and AP mice after administration of 100% GBBC. Hues of colors (from dark green (bottom scale) to faded red (top scale)) are represented by mean values.

Quantitative biochemical examinations provide an accurate assessment of illness development and hence aid in a better understanding of the disease process. Here, measurement of creatine kinase, amylase, and lipase activity showed that there was a strong relationship between the administration of 100% GBBC (po, 100 µL/mouse) and a reduction in inflammation in AP mice (Figure 3). In contrast, administration of GBBC did not notably decrease aspartate transaminase and alanine transaminase levels in AP mice. Moreover, there were no statistically significant differences between healthy mice, AP mice, and AP mice treated with GBBC in the following parameters: glucose, creatinine, urea, total protein, and calcium.

### 3.4. FFAR1 and FFAR4 Are Highly Expressed in the Mouse Pancreas

**Figure 4:**
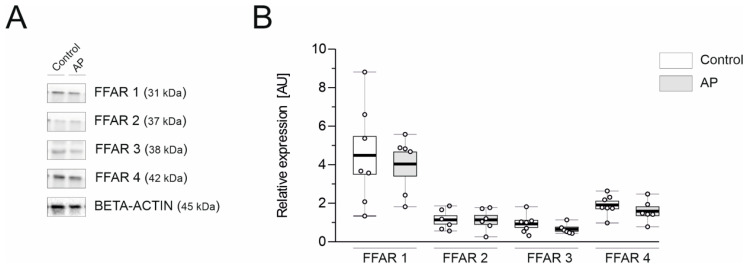
Representative western blots demonstrating the expression of all analyzed proteins—free fatty acid receptors (FFARs) (**A**) and changes in the protein concentration of FFARs (**B**) in the pancreas of healthy mice and mice with evoked acute pancreatitis. Beta-actin was used as a loading control for Western Blot to normalize the levels of protein. Data presented as raw data (circles) and means (horizontal bolded lines) with SEM (boxes) (**B**); *n* = 6–7.

As colostrum mainly consists of FAs, immune cells (as lymphocytes), and many antibodies, such as IgA, IgG, and IgM, we hypothesized that GBBC might exert an anti-inflammatory effect with the active involvement of FFARs. Therefore, we performed Western blot analysis in healthy controls and mice with AP to characterize the expression of FFAR proteins. FFAR1 had the highest expression both in control and AP mice (4.49 ± 0.99 and 4.04 ± 0.62 AU, respectively; Figure 4). FFAR4 was the second most highly expressed FFAR in the mouse pancreas (1.91 ± 0.20 and 1.59 ± 0.23 AU for control and AP mice, respectively; Figure 4). Notably, no significant differences in individual FFAR expression were found between control and AP mice.

### 3.5. The Anti-Inflammatory Activity of GBBC in L-Arginine-Induced AP in Mice Could Depend on FFAR1 and FFAR4

**Figure 5:**
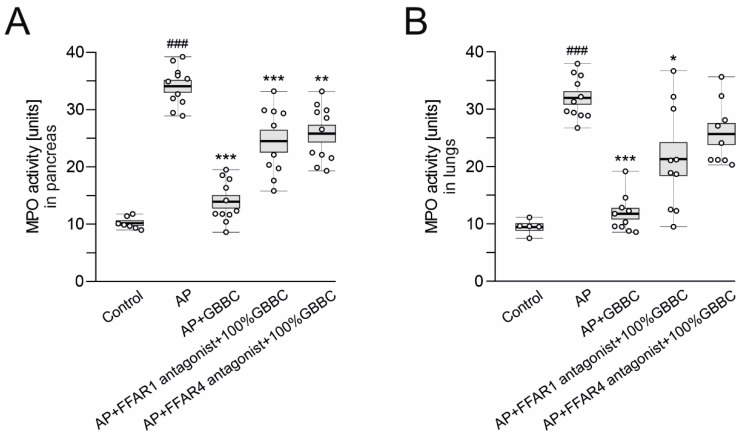
MPO activity level in pancreas (**A**) and lungs (**B**) of healthy mice, mice with evoked acute pancreatitis (AP), and mice with AP after administration of 100% GBBC and FFAR1 or FFAR4 antagonist. Data presented as raw data (circles) and means (horizontal bolded lines) with SEM (boxes). Significance estimated with the use of one-way ANOVA followed by Dunnett’s post hoc test. # (hash) denotes comparison to control. * (asterisk) indicates comparison to AP. *n* = 4–8; * denotes *p* < 0.05; ** denotes *p* < 0.01; ###/*** denotes *p* < 0.001.

Administration of an FFAR1 antagonist followed by treatment with 100% GBBC (po, 100 µL/mouse) significantly diminished the anti-inflammatory effect of GBBC, evidenced by MPO activity; of note, a similar inhibition of the anti-inflammatory effect of GBBC was observed during the administration of an FFAR4 antagonist (34.10 ± 1.04 U vs. 13.90 ± 1.09 U vs. 24.50 ± 1.95 U vs. 25.80 ± 1.47 U for AP vs. AP + 100% GBBC vs. AP + FFAR1 antagonist + 100% GBBC vs. AP + FFAR4 antagonist + 100% GBBC, respectively; Figure 5A).

Regarding MPO activity in the lungs, the anti-inflammatory effect of GBBC was also diminished by the FFAR1 antagonist (32.00 ± 1.11 U vs. 11.80 ± 0.93 U vs. 21.40 ± 2.87 U for AP vs. AP + 100% GBBC vs. AP + FFAR1 antagonist + 100% GBBC vs. AP, respectively; Figure 5B). The effect of the FFAR4 antagonist on GBBC action was also observed and was non-significant while compared to AP mice (25.70 ± 1.84 U vs. 32.00 ± 1.11 U, respectively; Figure 5B).

### 3.6. Morphological Analysis Revealed Decreased Severity of Pancreatitis after GBBC Treatment in AP Mice

#### 3.6.1. H&E Staining

**Figure 6:**
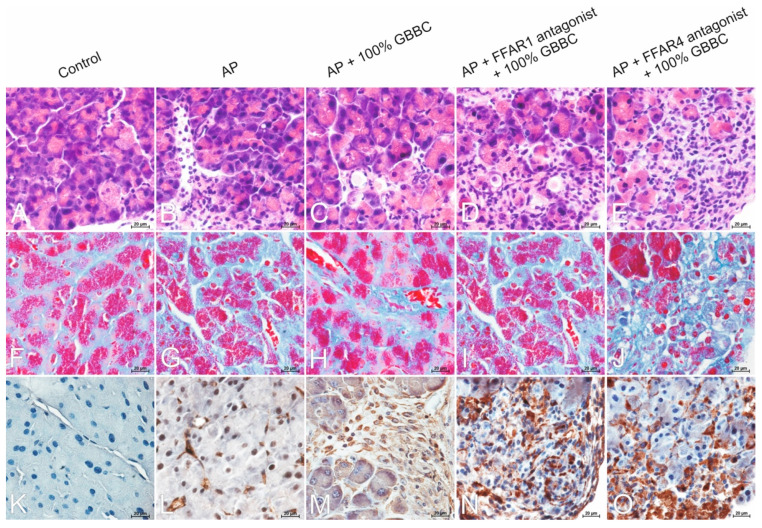
Microphotographs of the pancreas. H&E staining: control group—normal architecture of the exocrine pancreas with functional acinar cells rich in zymogen granules (**A**); acute pancreatitis (AP) group—diffuse inflammatory infiltrates in periacinar area with the predominance of neutrophils (**B**); AP + 100% ghee butter from bovine colostrum (GBBC) group—acinar cell vacuolization, minimal atrophy of acinar cells with no evidence of inflammatory cell infiltrates (**C**); AP + FFAR1 antagonist + 100% GBBC group—dissociation and moderate atrophy of acinar cells (**D**); AP + FFAR4 antagonist + 100% GBBC group—dissociation of acinar cells, strong remodeling of the stroma, and marked atrophy of acinar cells (**E**). Masson’s trichrome staining: control group—minimal collagen deposition as a delicate framework for acinar cells (**F**); AP group—minimal collagen deposition in inflamed and edematous stroma (**G**); AP + 100% GBBC group—minimal collagen deposition between well-compacted acinar cells (**H**); AP + FFAR1 antagonist + 100% GBBC group—moderate collagen deposition in periacinar area and between individual acinar cells (**I**); AP + FFAR4 antagonist + 100% GBBC group—marked deposition of collagen between atrophied acinar cells (**J**). Immunoreactivity of α-SMA protein: control group—negative reaction (**K**); AP group—positive reaction in individual myofibroblast-like cells (**L**); AP + 100% GBBC group—moderate positive cytoplasmic reaction in migrating myofibroblast-like spindle-shaped cells penetrating periacinar spaces (**M**); AP + FFAR1 antagonist + 100% GBBC group—strong immunohistochemical reaction in myofibroblast-like cells (**N**); AP + FFAR4 antagonist + 100% GBBC group—strong immunohistochemical reaction in numerous myofibroblast-like cells (**O**). Nikon’s Eclipse E600 light microscope (Nikon Instruments Inc., Tokyo, Japan) was used to examine tissue samples. Microphotographs were taken with a digital imaging system that included a microscopy digital camera (Nikon DS-Fil, Nikon Instruments Inc., Tokyo, Japan) and image analysis software (NIS-Elements BR-2.20, Laboratory Imaging, Praha, Czech Republic); 400× magnification.

The control group’s H&E-stained pancreas tissue slices revealed no microscopical alterations when examined histopathologically (Figure 6A). In contrast, acinar cell dissociation, disruption of pancreas histoarchitecture, acinar cell vacuolization and edema, moderate acinar cell atrophy, and significant neutrophil infiltration were observed in the L-arginine-treated group (Figure 6B). There was no evidence of inflammatory cell infiltrates in the periacinar area and minimal atrophy of acinar cells in the AP group treated with 100% GBBC (Figure 6C). Moreover, administration of the FFAR1 antagonist before GBBC emphasized the moderate dissociation and atrophy of acinar cells (Figure 6D), while administration of the FFAR4 antagonist followed by 100% GBBC revealed a pronounced process of the dissociation of acinar cells and strong remodeling of the exocrine portion of the pancreas along with the marked atrophy of individual acinar cells (Figure 6E).

#### 3.6.2. Masson’s Trichrome Staining

Collagen fibers in the pancreas were detected using Masson’s trichrome staining. The amount of collagen deposited in the extracellular matrix by fibroblasts is a well-managed equilibrium between collagen production and collagen catabolism in the pancreas. In the AP group, there was minimal collagen deposition in inflamed and edematous stroma (Figure 6G). In the AP + 100% GBBC group, there was minimal deposition of collagen fibers between well-compacted acinar cells (Figure 6H), similar to the control group (Figure 6F). In the AP group treated with the FFAR1 antagonist and then 100% GBBC, there was moderate collagen deposition in periacinar area (Figure 6I). Interestingly, after administration of the FFAR4 antagonist followed by treatment of the AP group with 100% GBBC, marked collagen fiber deposition was observed between individual acinar cells (Figure 6J).

#### 3.6.3. Immunohistochemical Reaction with Anti-α-SMA

There was a negative immunohistochemical reaction in the exocrine portion of the pancreas in the control group (Figure 6K). Immunoreactivity for α-SMA protein in individual activated myofibroblast-like cells was detected in the AP group (Figure 6L). A moderate positive cytoplasmic reaction in migrating myofibroblast-like spindle-shaped cells penetrating periacinar spaces was noted in the AP group treated with 100% GBBC (Figure 6M). A strong immunohistochemical reaction was detected in myofibroblast-like cells of the AP group treated with the FFAR1 antagonist and then 100% GBBC (Figure 6N). Similarly, strong immunoreactivity for α-SMA protein was detected in numerous myofibroblast-like cells of the AP group treated with the FFAR4 antagonist followed by 100% GBBC (Figure 6O).

## 4. Discussion

AP is caused by several factors, including bacterial and viral infection, pancreatic duct blockage, abdominal trauma or surgery, higher blood calcium levels, and abnormally high triglyceride levels. The most common cause, on the other hand, is excessive alcohol use. These factors appear to prompt digestive enzymes (amylase, lipase, and creatine kinase) to act on the pancreas, resulting in edema, bleeding, and injury to the pancreatic blood vessels. Typically, AP symptoms are severe and necessitate medical attention. Pancreatic cysts, abscesses, and pancreatic fluid leakage into the stomach can occur if the condition is left untreated for a long time, resulting in life-threatening peritonitis or long-term medical complications. Furthermore, after a history of recurrent attacks of AP, CP develops over several years.

Management of AP is still largely supportive and, undoubtedly, new treatment strategies are critically needed to alleviate AP symptoms. Here, using the L-arginine animal model, we investigated the potential anti-inflammatory properties of GBBC in the treatment of AP as well as attempting to characterize the role of FFAR.

The AP model utilized in the study has several advantages: it is inexpensive, technically straightforward to execute, and only requires *ip* injections. The method of induction is non-invasive and does not require anesthesia or surgery. Furthermore, it accurately reproduces most human AP morphological features. One distinction between human and mouse L-arginine-induced AP is that human disease has a patchy distribution, whereas mouse AP does not. Unfortunately, the severity of the condition is difficult to manage (especially in mice) when given a high dose of L-arginine; in consequence, systemic toxicity and animal death may occur, which is a limitation of the model [29,30].

The results of our study indicated that GBBC may have a strong anti-inflammatory effect in the mouse model of L-arginine-induced AP, as evidenced by decreased MPO activity (in the pancreas and lungs) and selected biochemical markers (in blood serum), as well as—most notably—histological abnormalities in the pancreatic tissue (H&E staining, Masson’s trichrome staining). AP appears as an inflammatory cell infiltrate with functional and/or structural acinar cell destruction, as well as duct cell necrosis in rare cases, on a microscopic level. Our morphological analysis revealed that GBBC treatment after AP induction reduced inflammatory cell infiltrates in the periacinar area and resulted in minimal acinar cell atrophy.

Of note, lowered lipase activity in the pancreas of the AP group treated with GBBC compared with AP mice emphasized the possible involvement of FFARs. This was initially confirmed by the administration of FFAR1 and FFAR4 antagonists.

FFAR1 and FFAR4 bind medium- to long-chain, saturated, and omega-3 unsaturated FAs [31]. Both receptors are under increasing investigation for their roles in diabetes and, more recently, also cancer, but most importantly in inflammation processes. Hidalgo et al. [32] discovered that oleic acid increased ERK1/2 phosphorylation, superoxide production, CD11b expression, and matrix metalloproteinase-9 release in FFAR1-expressing bovine neutrophils in an intracellular Ca^2+^ concentration ([Ca^2+^]_i_)-dependent manner. Furthermore, the authors demonstrated that GW9508, a partial agonist of FFAR1, induced intracellular calcium mobilization and ERK2 phosphorylation. According to Fujita et al. [33], GW9508 activation suppressed the induction of cytokines and chemokines in keratinocytes and reduced allergic inflammation in the skin. Furthermore, Nagatake et al. [34] discovered that 17,18-epoxyeicosatetraenoic acid, a metabolite of EPA, acted as an FFAR1 ligand and exhibited antiallergic and anti-inflammatory effects by inhibiting neutrophil mobility in mouse and cynomolgus macaque contact hypersensitivity models.

FFAR4 is found in high concentrations in the adipose tissue and proinflammatory macrophages, implying that it plays important roles in these cells. In the presence of ligands such as DHA and EPA, Oh et al. [35] demonstrated that FFAR4 agonists have anti-inflammatory functions in monocytic RAW264.7 cells and primary intraperitoneal macrophages. Using an in vitro system, the authors also demonstrated that the mechanism underlying the DHA-mediated anti-inflammatory response, such as inhibition of both the TLR and TNF-signaling pathways, involved inhibition of TAK1 phosphorylation via an aarres-tin-2/TAK1 binding protein 1 (TAB1)-dependent effect. Furthermore, in vivo investigations demonstrated that omega-3 FA administration reduced tissue inflammation generated by a high-fat meal (60 kcal % fat) in wild-type mice, enhancing systemic insulin sensitivity; however, similar effects were not found in FFAR4-deficient mice [36]. In 3T3-L1 adipocytes, Yamada et al. [37] discovered that EPA reduced palmitate-induced increases in inflammatory gene expression and NF-ϰB phosphorylation, and that silencing FFAR4 via siRNA diminished EPA’s anti-inflammatory effects. Additionally, they demonstrated that EPA supplementation reduced inflammatory signal transduction and the macrophage phenotype in the adipose tissue of mice fed a high-fat, high-sucrose diet. Zhao et al. [38], on the other hand, used immunohistochemical analysis to show that FFAR4 is found in the pancreas of both humans and rats, and it colocalizes with CD68 (a macrophage-specific marker), CD34, and CD117 (interstitial cell markers in the pancreas). Furthermore, Konno et al. [39] studied the expression and activity of FFAR4 in human eosinophils in vitro using synthetic agonists and reported that stimulating FFAR4 with a synthetic ligand decreased spontaneous apoptosis and enhanced the release of interleukin IL-4. Furthermore, positive results for synthetic FFAR compounds were obtained during early clinical trials, implying that future research will expand FFARs’ therapeutic potential [31]. Moreover, under the influence of TGF-β (transforming growth factor-β) secreted by epithelial or inflammatory cells, fibroblasts are activated and migrate to injured areas in the pancreas [40]. As a result of transformation (fibroblast to myofibroblast transition, FMT), fibroblasts change their phenotype, manifesting the morphological features of myofibroblasts and synthesizing smooth muscle actin as a cytoskeletal component. Myofibroblasts play a key role in the remodeling of the pancreatic stroma, including the process of fibrosis leading to CP. In our study, there was evidence of FMT in all AP groups with different immunoreactivity of α-SMA (Figure 6L–O). α-SMA reactivity and the degree of collagen deposition affect the course of AP during matrix remodeling.

This is, to the best of our knowledge, the first study to suggest that GBBC may be beneficial during the management of AP, and we believe that the effect might be dependent on FFARs. Certainly, further translational research into GBBC and FFARs may be necessary to validate their therapeutic potential for treating AP and other inflammatory diseases. However, our study gives strong support for bovine colostrum and its derivatives as an important source of factors (e.g., FAs, lactoferrin, casein, immunoglobulins) necessary to support cell and tissue development and repair. Moreover, since GBBC demonstrates anti-inflammatory effects in the L-arginine mouse model of AP, it might be considered a first-in-class functional product (additive) in the management of AP.

## 5. Study Limitations

If delving more deeply into the topic, one might consider adding experiments employing FFAR2 and FFAR3 antagonists. However, low expression of these two receptors in the pancreas of healthy mice and mice with AP (Figure 4) along with the composition of FAs in GBBC do not advocate for such experiments. Moreover, as an FFAR3 antagonist is not commercially available, we would not be able to fully investigate the involvement of this receptor in the anti-inflammatory effect of GBBC in murine L-arginine-induced AP. In consequence, experiments using FFAR knockout mice, to fully elucidate the involvement of FFARs in GBBC action in AP, could be of great importance for our understanding of the effects observed herein. However, these would require substantial financial and conceptual input taking into consideration the current state of the art in the field.

## Data Availability

The data presented in this study are available on request from the corresponding author.
